# Lumping Sexes and Splitting Genders

**DOI:** 10.1007/s10508-025-03338-5

**Published:** 2025-10-27

**Authors:** Logan Trevino-Fica, S. Marc Breedlove

**Affiliations:** 1 Sociology Department, Michigan State University, East Lansing, MI USA; 2 Neuroscience Program, Michigan State University, East Lansing, MI 48824 USA

Scientists have always been in the business of lumping or splitting. To make sense of a diverse world, humans categorize what we encounter in nature. Supposedly, Adam’s first task was to distinguish and name the various animals. A difficulty with classifying animals is that all species are in some ways alike (they use DNA to pass on traits, exploit the Krebs cycle for energy, have digestive systems, etc.), yet each individual member of any population is in some ways unique, thanks in part to the immense diversity provided by the recombination of DNA. Even monozygotic twins differ from one another thanks to pervasive environmental effects on the body and brain. Extremists could lump all animals by their commonalities to say there is only one animal species on Earth or split them by their distinctive qualities to say every individual is a unique species.

As such, early naturalists continuously debated whether any pair of specimens were sufficiently alike to be lumped into one species or sufficiently different to be split into two. The debate might never have ended because it relied on subjective judgment about whether a difference (and, depending on how hard one looks, there are always differences) was different enough. Then in the mid-twentieth century, an objective criterion for distinguishing species was offered. If individuals from the two populations could produce fertile offspring, they were a single species, but if no fertile offspring were produced, they were separate species. Arguments then shifted to determining how many subspecies there are, allowing debate between lumpers and splitters to continue without any risk of resolution.

Splitting versus lumping applies to any categorization process, including the one at hand: How many sexes and genders are there?

## The First Cut Is the Deepest

Immediately, we are met with the question of whether to lump the concepts together, as suggested by their interchangeable usage in many corners (where one suspects writers consider genteel “gender” more polite than brazen “sex”) or to split them into two separate concepts. We firmly advocate the latter, considering sex to refer to population-level biological distinctions rooted in objectively observable criteria (whether or not they have, in fact, been objectively described in the past) with an evolutionary history from ancestral species, while gender is a culturally constructed notion of characteristics and behaviors that people should, or usually do, display to fit categories such as man, woman, or, in many cultures, some other category such as two-spirit people. Our advocacy of this first split is not meant to suggest that one is more important or valuable than the other. Rather, we think that distinguishing sex from gender is useful, medically and scientifically (National Academies of Sciences, Engineering, and Medicine, 2022), despite the recent US Executive Order 7301 forbidding study of gender apart from two and only two sexes.

## How Many Sexes Are There?

From a biological perspective, there is indeed a fundamental dichotomy for every known animal species: the size of gametes. There are only two ways natural selection has hit upon for animals to sexually reproduce: either producing large gametes (ova) or small gametes (sperm). Humans have designated those that produce ova as female and those that produce sperm as male. In hermaphroditic species, each individual produces both, but no species has been found to use exclusively medium-sized gametes. Individuals may have tried making medium-sized gametes (spova? overms?), but they left no descendants, so that appears to be an evolutionary dead end for metazoans on our planet. Thus, evolution has imposed a strict dichotomy on animal gametes and we find this distinction inviolate. However, after that, characteristics associated with these two kinds of gametes often present as a spectrum.

For example, each type of vertebrate gonad produces a preponderance of particular hormones, such as androgens like testosterone, or estrogens like estradiol, but there is no hormone produced solely by either gonad. Indeed, in rodents, it is the estrogenic metabolites of testosterone that actually masculinize the brain. So, even at the population level, there are no “male hormones” or “female hormones” for any vertebrate species.

The blurring of any dichotomy past the level of gametes is especially prominent when you move beyond generalizations about populations to individuals in that population. For example, most gonads are specialized to produce either ova (ovaries) or sperm (testes), but some rare individuals have an ovotestis. Given only two kinds of gametes, sex could be defined based on whether the individual has the physiology to reproduce with eggs or with sperm or with both. However, in every population, there are infertile individuals who, for one reason or another, cannot reproduce at all. Then you could say, “Okay, their structures to produce gametes and/or distribute them may not be functional, but one could still categorize them based on whether their structures are those normally associated with one gamete or the other.” Even here, some individuals with differences of sex development/disorders of sexual differentiation (DSDs) have a “mosaic” phenotype: Some parts of their body would ordinarily support reproducing via sperm, while others would typically support reproducing via eggs. We are again faced with whether to split or lump.

So, while there may be only two biological sexes on a population level, a given individual may have some physical characteristics typically found only in males, with other characteristics typically only found in females. One might take refuge by saying that the vast majority of people have structures normally associated with only one sex, but that probably depends on how thoroughly you examine the structures and where exactly you place the boundary between masculine and feminine. Which means we are left, like early naturalists without an objectively verifiable definition of “species,” with a perpetually unresolvable debate about how to classify a person’s sex or if we should even try. On the other hand, DSDs and individuals with physical characteristics from both sexes are relatively rare (how rare depending on one’s definitions). One could argue that, if a majority of people can be firmly categorized as possessing ova or sperm and the biological traits associated with each that is a “good enough” definition of sex.

We cannot address whether such a dichotomous categorization of people is correct, but one could ask whether it is useful. It is certainly useful in medicine to run Pap smears only in some people and digital exams of the prostate in others (we will put aside those rare people with DSDs who have both). For the majority of people, their external characteristics provide a good guess as to whether they will also have a uterus or a prostate. Beyond knowing what to probe during annual exams, there are considerable sex differences in the occurrence of disorders and in the most effective doses of medicines, which has been documented thoroughly (Prendergast et al., 2014; Zucker & Prendergast, 2020), but which have traditionally been overlooked because of the casual misogyny of past science to consider only males worthy of interest. It would be unfortunate if the sexism in medical science that neglected female health issues for centuries remained through a willful blindness to sex entirely. Likewise, it would be unfortunate to remain ignorant of individuals who wish to remain outside of this dichotomy (Haghighat et al., 2023). Lumping the sexes is to the detriment of health care for all.

The greatest risk of keeping a person’s apparent sex as a criterion for medical care would be an overly zealous application on the individual level, but this is generally avoidable with some thought. Most practitioners would understand it is ridiculous to perform a prostate exam on someone based on the presence of a penis over the presence of a prostate.

## How Many Genders Are There?

For gender, the case is quite different. The nature of human social behavior means that the (assumed) presence of one gamete or the other brings along additional expectations of comportment, appearance, and attitude that extend beyond the mating and courtship behaviors seen in other animals. The social categories associated with gametes have material implications for the realities of both individuals and societies at large. Gender as a term captures the social distinction that explains how female and male translate to women/girls and men/boys and the expectations for how one ought to behave based on placement in those categories. Gender as a social rather than biological distinction, separate but normatively associated with sex, allows us to explain why these expectations vary so greatly across cultures and can change across time within a given culture, even though the basic biology of sexual reproduction was fixed long before our species arose. It also explains why some cultures, but not others, recognize more than two genders.

If a culture can recognize three genders, then why not more? Fausto-Sterling (2000) provocatively suggested there are five genders, and offered a cogent disquisition of how they might differ from one another (see also Goymann et al., 2023). But, as in all cases where lumpers and splitters engage their efforts, it is not clear whether there might not be well more than five. Because most people display a mix of behaviors that are typically associated with men and others that are typically associated with women, we suspect that, if one measured those gradations finely enough, no two people would be exactly alike, any more than monozygotic twins are. In that case, an extreme splitter could claim that there are as many genders as there are people.

Counteracting an infinite spectrum of genders is that, as a social construct, culture also has a say in how many genders are generally recognized and that may impose a limit. Again, it might depend on how different two behavioral profiles must be to be considered separate genders rather than different points on the range of a single gender. Are there any cultures that recognize more than five genders? Even if there are not any examples known at present, who is to say that our culture would not recognize a dozen some day? It is the nature of culture to change over time, especially over generations. But presumably there is some upper limit to the number of genders a given society can recognize to be widely accepted and applied to interpersonal relations. Once a society recognizes a gender, it will also provide a guideline for how a person of that gender should behave themselves within some range of variation; otherwise, we will be back to each person having a unique gender, which would be equivalent to there being no genders at all. However, we ask, would it be bad thing if government kept no notice of any given citizen’s gender?

To the question of how many there are now: In the USA, most people recognize two, and certainly the federal government recognizes only two (Exec. Order No. 14,168, 2025; Public Religion Research Institute, 2023). Individuals are expected to conform within the two categories of female-woman and male-man. However, were there no debate there would be no need for this commentary and, in some places, there is legal recognition of at least three genders (though called “sexes”) and further recognition of gender as distinct from sex through mechanisms like self-identification (Michigan Department of State, 2021). In other places, people argue for an even greater number of individual genders and terminology associated with them (Cassian, 2024), although they have yet to see widespread cultural recognition in the same way three-gender categorization has.

Cultures evolve, too. Historically, gender has been associated with myriad behaviors and traits no longer necessary for “proper” gender identification today. The length of someone’s hair and whether they wear pants is no longer as clear-cut an indicator of their gender as it might have been a century ago. Homosexual attraction no longer widely indicates “gender inversion” as a pathology (Brown, 1957), despite heterosexuality being considered a critical aspect of “normal” gender development for decades (Farvid, 2015). Who is to say that American categories of gender cannot or would not entirely divorce themselves from male and female, or man and woman, as they have from pants, hairstyles, and sexual attraction? Again, we ask, would that be such a bad thing?

## Conclusion

To the question “how many sexes/genders are there?,” we offer two conclusions. The first is that, on a population level, for all sexually reproducing animals on this planet, there are two sexes as determined by size of the gametes they produce. When one digs further than that, there are finer gradations and differences both between species and within them, but broadly, we find it most useful to divide the sexes by gametes. Importantly, the characteristics associated with one gamete or the other at the population level often present as a continuum and there have always been some individuals bearing both female- and male-typical characteristics. Even so, we see no utility in trying to devise complex guidelines to split sexes into more than two categories, as it could be done, in theory, ad infinitum.

The second is that gender acts as a social category associated with sex, wherein individuals have expectations placed on them based upon their gender category. Gender, as a construct, is malleable and capable of changing over time and location, and, in the USA, there are efforts to change the current two-category system to a three-category system, or more. What we posit to the reader for the final time is: Would that be such a bad thing? Put another way, what compelling interest would an egalitarian government or society have to inquire about any given citizen’s sex, much less their gender? Medical professionals may need to know an individual’s sex to provide optimal care, but what difference would (or should) it make for driving a car, paying taxes, obeying laws, or enjoying the rights of citizenship? What difference would (or should) it make to the kinds of jobs, education, and resources people have access to? We may not live in that world now, but we could live in that world in the future—a future invested in seeing all people as individuals first, not by their sex or their gender. A world, in some way, comprised of splitters.
